# RC Bridge Oscillation Memristor Chaotic Circuit for Electrical and Electronic Technology Extended Simulation Experiment

**DOI:** 10.3390/mi14020410

**Published:** 2023-02-09

**Authors:** Gang Dou, Yongcheng Zhang, Hai Yang, Mingqiao Han, Mei Guo, Wendong Gai

**Affiliations:** 1 The College of Electrical Engineering and Automation, Shandong University of Science and Technology, Qingdao 266590, China; 2 Faculty of Science and Engineering, University of Nottingham Ningbo China, Ningbo 315100, China

**Keywords:** memristor, chaotic circuit, RC bridge oscillator, electrical and electronic technology experiment

## Abstract

The fourth basic circuit component, the memristor, has been proposed for a long time, but it is not mentioned in the experiment teaching system of Electrical and Electronic Technology. In this paper, an RC bridge oscillation chaotic circuit based on memristor is designed to solve this problem. The dynamical behavior of the circuit system is analyzed using Lyapunov exponents spectrum, bifurcation diagram, phase portrait and Poincaré map. A series of complex dynamical behaviors such as symmetric single-scroll coexistence, asymmetrical single-scroll coexistence, symmetric double-scroll coexistence and asymmetrical limit–cycle coexistence exist in the circuit system. This research plays a critical role in enriching students’ knowledge and improving the experiment teaching system of Electrical and Electronic Technology.

## 1. Introduction

In 1971, Chua predicted the existence of the memristor as the fourth circuit elements except resistance, inductance, and capacitance [1]. In 2008, researchers at Hewlett-Packard Labs developed the first physical memristor element, which drew academic attention to memristors [2]. In 2011, Torrezan and Strachan et al. tested a metal-tantalum oxide-metal structured memristor integrated in an experimental device with a 20 GHz bandwidth, and found that it had sub-second switching characteristics [3]. In 2020, Kim et al. found that a memristor based on a quasi-two-dimensional structure of chalcogenide material as a semiconductor dielectric has a more desirable switching ratio and stability [4]. In 2021, Almadhoun et al. developed a bipolar memristor that can maintain a resistive on/off state for up to $10^{5}$ s [5]. With the development of neural networks [6,7,8], memristor has become one of the important ways to realize neural network circuits [9,10]. Due to its ability to hysterically change its resistance in response to previously applied electrical stimuli [11,12], the memristor has attracted considerable interest in various hypothetical applications, such as nonvolatile memories [13,14], logic gates [15,16,17], hybrid logic/memory circuits [18,19,20], and neuromorphic computing [21,22,23,24,25]. In addition, the unique features of this device have stimulated interest in generating nonlinear and chaotic dynamics [26,27]. In recent years, the research and application on chaotic systems have become more and more abundant [28,29,30,31]. By introducing memristors with different nonlinearities into existing circuits, a large number of circuits based on memristor have been proposed and very fruitful results have been obtained [32,33,34,35,36,37,38]. For example, complex dynamic behaviors have been discovered in various memristor-based circuits, including multistability [39], transient chaos [40], and hyperchaos [41]. Although the research of memristor is more and more abundant now, there are few applications in the experimental teaching system of Electrical and Electronic Technology. This experiment aims to enrich students’ experience and improve their ability to adapt to society [42]. Therefore, it is necessary to apply memristor to teaching systems.

Most of the memristors used in current chaotic circuits are implemented by analog circuit and FPGA [43]. In the paper, a flux-controlled mathematical model of physical SBT memristor is used [44,45]. In this work, the physical memristor is used and the program design is simplified to adapt to the experimental teaching. This experiment is implemented by simulation, in order to analyze its dynamic behavior conveniently. An RC bridge oscillation chaotic circuit based on physical memristor is proposed, and its dynamic behaviors, especially the coexistence of multi-dynamics, are analyzed by means of Lyapunov exponents spectrum bifurcation diagram and phase portraits.

The rest of the paper is organized as follows: Section 2 presents the mathematical model of flux control of physical memristor and RC bridge chaotic circuit, and the equation of state for the circuit was established by the voltage–current relationship of circuit elements and Kirchhoff’s circuit law. Section 3 analyzes the stability of the equilibrium point, the influence of the initial state and circuit parameters on the system dynamics through the Lyapunov exponents spectrum, and the bifurcation diagram. Section 4 focuses on the effect of flux change on the circuit. Finally, the conclusions are given in Section 5.

## 2. Chaotic Circuit Based on Quadratic Smooth Model of Memristor

In our previous work, we prepared the SBT memristor [44]. As a new circuit element, memristor represents the connection between charge and flux. A quadratic smoothing model is applied in this paper to describe this connection [45].

$$q=f\varphi \left|\varphi \right|+g\varphi +h$$

$$\left\{\begin{array}{c} u\left(t\right)=\frac{d\varphi \left(t\right)}{dt}\\ i\left(t\right)=\frac{dq\left(t\right)}{dt} \end{array}\right.$$

$$W\left(\varphi \left(t\right)\right)=\frac{dq\left(t\right)}{d\varphi \left(t\right)}$$

$$W\left(\varphi \left(t\right)\right)=j+k\left|\varphi \left(t\right)\right|$$

where $\varphi \left(t\right)$ and $q\left(t\right)$ represent magnetic flux and charge, respectively, and $i\left(t\right)$ and $u\left(t\right)$ represent the current flowing through the memristor and the voltage at both ends.

**Figure 1:**
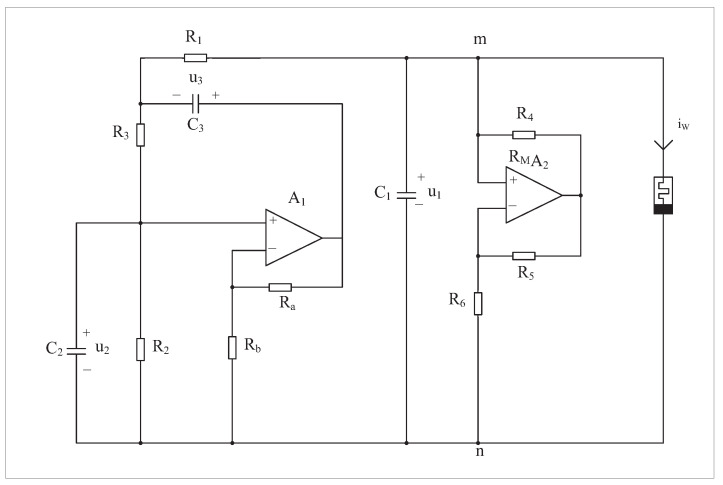
Chaotic circuit based on a quadratic smooth model of an SBT memristor.

A chaotic circuit including a second-order RC bridge oscillator, capacitor, negative resistor and SBT memristor is constructed as shown in Figure 1. The RC oscillating bridge circuit can generate sinusoidal waves with adjustable frequency, which lays out the condition for the subsequent test. Coupling capacitors can connect the front and rear circuits. Negative resistance can play a controlling role. The existence of memristors is one of the conditions of chaos. Setting $R_{2}=R_{3}$, $C_{2}=C_{3}$ and amplification $A_{u}$ of op-amp $A_{1}$ is as follows:

$$A_{u}=1+\frac{R_{a}}{R_{b}}$$

The second-order RC bridge oscillator satisfies the oscillation condition. The oscillation circuit is coupled with the memristor through $C_{1}$. In previous studies, the mathematical model of the memristor had been proposed [45], and the quadratic smooth model of the memristor was obtained based on experimental measurements. The model of the memristor is as follows:

(1) $$\left\{\begin{array}{c} W_{2}\left(\varphi \left(t\right)\right)=A+B\left|\varphi \left(t\right)\right|\\ \frac{d\varphi \left(t\right)}{dt}=u\left(t\right) \end{array}\right.$$

where A=0.03S, B=0.0614S/Wb, the current $i_{W}$ flowing through the SBT memristor is expressed as:

$$i_{W}=u_{1}\cdot W\left(\varphi \left(t\right)\right)$$

The equivalent negative resistance $R_{mn}$ between nodes *m* and *n* is:

$$R_{mn}=-\frac{R_{4}R_{6}}{R_{5}}$$

The equation of state for the circuit shown in Figure 1 is:

(2) $$\left\{\begin{array}{c} \frac{du_{1}\left(t\right)}{dt}=\frac{1}{C_{1}}\left[\frac{Au_{2}-u_{3}-u_{1}}{R_{1}}-\frac{u_{1}}{R_{mn}}-u_{1}\cdot W\left(\varphi \left(t\right)\right)\right]\\ \frac{du_{2}\left(t\right)}{dt}=\frac{1}{C_{2}}\left[\frac{\left(A-1\right)u_{2}-u_{3}}{R_{3}}-\frac{u_{2}}{R_{2}}\right]\\ \frac{du_{3}\left(t\right)}{dt}=\frac{1}{C_{3}}\left[\frac{Au_{2}-u_{1}-u_{3}}{R_{1}}+\frac{\left(A-1\right)u_{2}-u_{3}}{R_{3}}\right]\\ \frac{d\varphi \left(t\right)}{dt}=u_{1} \end{array}\right.$$

The four state variables $u_{1}\left(t\right)$, $u_{2}\left(t\right)$, $u_{3}\left(t\right)$, $\varphi \left(t\right)$ in Equation (2) correspond to the voltage of the capacitor $C_{1}$, the voltage of the capacitor $C_{2}$, the voltage of the capacitor $C_{3}$ and the internal flux of the SBT memristor. Setting $\tau =\frac{t}{C_{2}R_{2}}$, $x=\frac{u_{1}}{E}$, $y=\frac{u_{2}}{E}$, $z=\frac{u_{3}}{E}$, $\omega =\frac{\varphi }{C_{2}R_{2}E}$, $R_{2}=R_{3}$, $C_{2}=C_{3}$, E=1V, $C_{2}R_{2}=1\;s$, the dimensionless dynamical system Equation (3) is obtained as follows:

(3) $$\left\{\begin{array}{c} \frac{dx}{d\tau }=a\left[b\left(Ay-z-x\right)-cx-x\cdot W\left(\omega \right)\right]\\ \frac{dy}{d\tau }=\left(A-2\right)y-z\\ \frac{dz}{d\tau }=b\left(Ay-x-z\right)+\left(A-1\right)y-z\\ \frac{d\omega }{d\tau }=x \end{array}\right.$$

where $a=\frac{C_{2}}{C_{1}}$, $b=\frac{R_{2}}{R_{1}}$, $c=\frac{R_{2}}{R_{mn}}$, $W\left(\omega \right)=0.03+0.0614\left|\omega \right|$.

When setting the initial states as (0.01, 0, 0, 0), a=40, b=0.15, c=−0.2, $A_{u}=3.2$, the numerical simulation yields four Lyapunov exponents as $LE_{1}=0.0123$, $LE_{2}=0.0027$, $LE_{3}=0.0350$, and $LE_{4}=-3.03$, which indicate that the system is chaotic.

**Figure 2:**
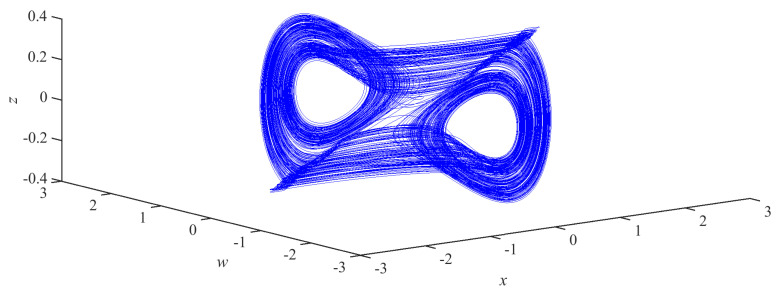
The three-dimensional phase portrait of the chaotic circuit system.

**Figure 3:**
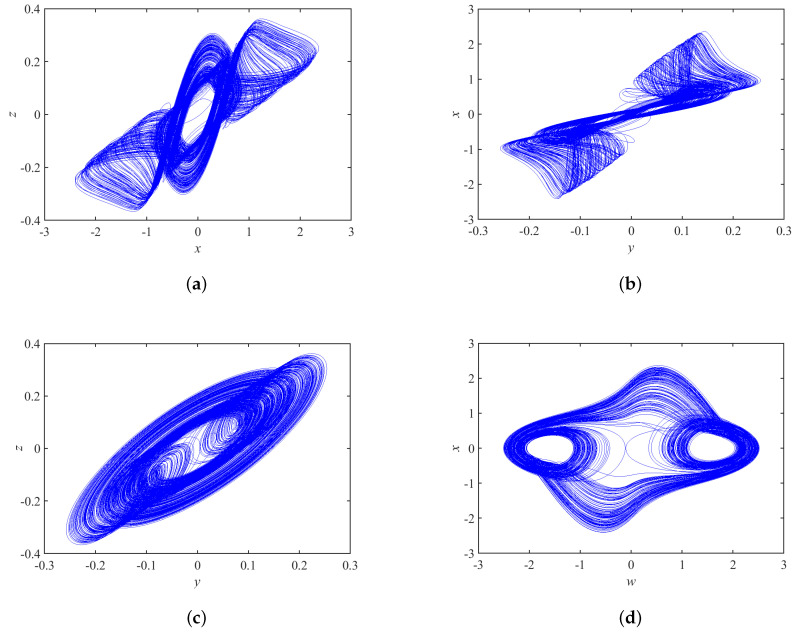
Phase portrait of the proposed chaotic system on (**a**) x–z plane, (**b**) y–x plane, (**c**) y–z plane, and (**d**) w–x plane.

The three-dimensional and two-dimensional phase portraits of chaotic circuit system are shown in Figure 2 and Figure 3, respectively. The circuit system shows a double-scroll attractor phenomenon at this time.

**Figure 4:**
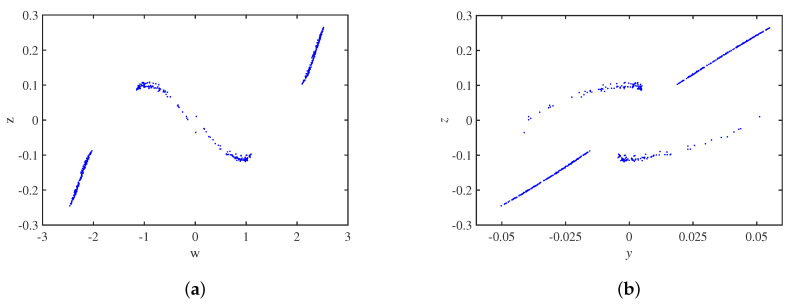
Poincaré maps: (**a**) y = 0; (**b**) x = 0.

The Poincaré maps on the two cross-sections y=0 and x=0 are in Figure 4, and the trajectory of the circuit system has a complex folding phenomenon.

**Figure 5:**
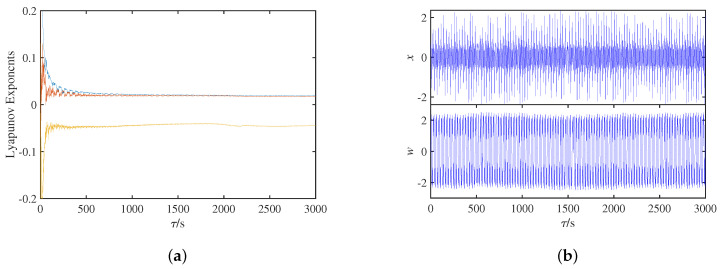
Dynamic behavior of circuit system with τ: (**a**) Lyapunov exponents spectrum; (**b**) time domain waveform.

Figure 5a shows the spectrum of Lyapunov exponents of the system in the time interval $\left[0,\;3000\right]$, where the fourth negative Lyapunov exponent is omitted for the convenience of observation. The maximum Lyapunov exponents are always positive, and the sum of all the Lyapunov exponents is always negative, which represents that the system has chaotic attractors. In Figure 5b, the time domain waveforms with respect to the state variables *x* and *w* are non-periodic irregular oscillations. In summary, it can be judged that the circuit system (Figure 1) has chaotic oscillation, and it is proved that the quadratic smooth model based on the SBT memristor can be used to construct chaotic circuits.

## 3. Dynamical Behavior of Chaotic System

### 3.1. Equilibrium Point and Stability Analysis

In Equation (3):

$$\frac{dx}{d\tau }=0,\frac{dy}{d\tau }=0,\frac{dz}{d\tau }=0,\frac{dw}{d\tau }=0$$

the set of equilibrium points of this system can be obtained as:

$$E=\left\{\left(x,\;y,\;z,\;w\right)\left|x=y=z=0,\;w=\varphi \left(0\right)\right.\right\}$$

where $\varphi \left(0\right)$ is an arbitrary constant, and the Jacobi matrix of the system is:

(4) $$J=\left[\begin{array}{cccc} -a\left(b+c+W_{2}\right) & abA & -ab & 0\\ 0 & A-2 & -1 & 0\\ -b & bA+A+1 & -b-1 & 0\\ 1 & 0 & 0 & 0 \end{array}\right]$$

The eigenvalue equation of the system Jacobi matrix is as follows:

$$\left|\lambda I-J\right|=\lambda \left(\lambda^{3}+A_{2}\lambda^{2}+A_{1}\lambda +A_{0}\right)=0$$

In Equation (4), $W_{2}=0.03+0.0614\left|\varphi \left(0\right)\right|$, the system parameters a=40, b=0.15, c=−0.2, $A_{u}=3.2$, and the three coefficients of the characteristic equation are obtained:

$$\left\{\begin{array}{c} A_{2}=2.456\left|\varphi \left(0\right)\right|-0.85\\ A_{1}=-0.1228\left|\varphi \left(0\right)\right|+0.44\\ A_{0}=3.1928\left|\varphi \left(0\right)\right|-2.84 \end{array}\right.$$

Referring to the Routh–Hurwitz stability criterion, the sufficient condition that all the characteristic roots of this circuit system have negative real parts is obtained as:

$$H_{k}=\left|\begin{array}{ccc} A_{0} & A_{2} & 0\\ 1 & A_{1} & 0\\ 0 & A_{0} & A_{2} \end{array}\right|>0$$

where k=1,2,3, then:

(5) $$\left\{\begin{array}{c} H_{1}=A_{2}>0\\ H_{2}=A_{0}>0\\ H_{3}=A_{2}A_{1}-A_{0}>0 \end{array}\right.$$

From Equation (5), the stability interval of the circuit system corresponding to $\varphi \left(0\right)$ is a null set, so the system is always unstable.

### 3.2. Dynamics Analysis with Initial Values

**Figure 6:**
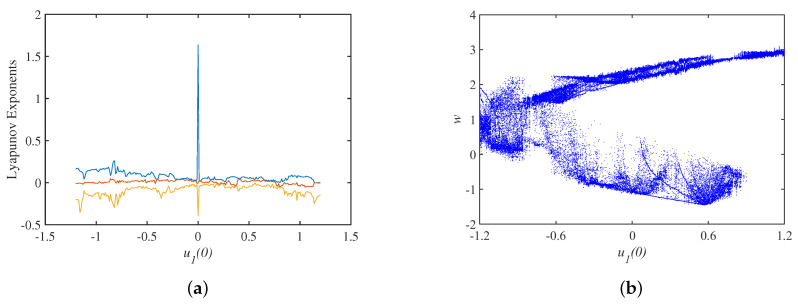
Dynamics analysis under the initial state $u_{1}\left(0\right)$: (**a**) Lyapunov exponents spectrum; (**b**) bifurcation diagram.

**Figure 7:**
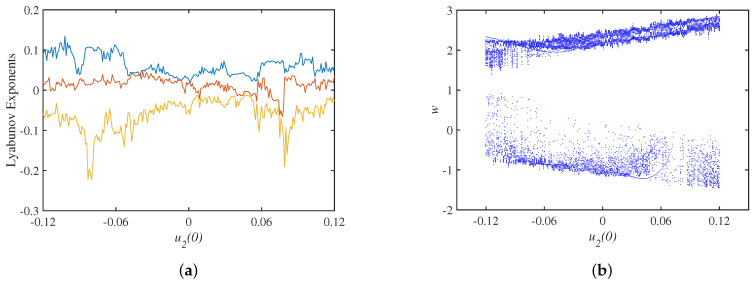
Dynamics analysis under initial state $u_{2}\left(0\right)$: (**a**) Lyapunov exponents spectrum; (**b**) bifurcation diagram.

**Figure 8:**
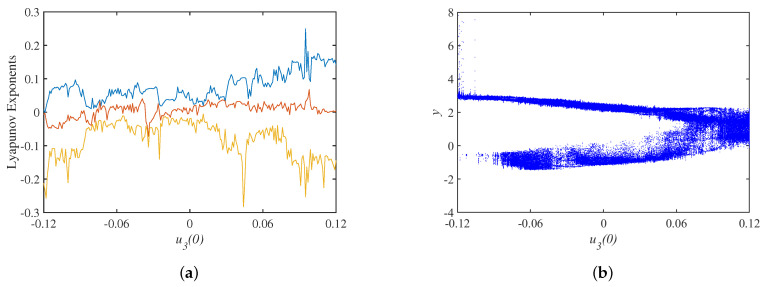
Dynamics with under initial state $u_{3}\left(0\right)$: (**a**) Lyapunov exponents spectrum; (**b**) bifurcation diagram.

Setting the parameter set $S=\left\{\left(a,\;b,\;c,\;A\right)\left|a=40,\;b=15,\;c=-0.2,\;A_{u}=3.2\right.\right\}$, The Lyapunov exponents spectrum with the initial state $u_{1}\left(0\right)$ is shown in Figure 6a, where the smallest Lyapunov exponents is omitted, and the bifurcation diagram corresponding to the y=0 cross section is shown in Figure 6b. Keeping the same parameter set, the spectrum of Lyapunov exponents and bifurcation diagrams with the initial state $u_{2}\left(0\right)$ are shown in Figure 7, and the spectrum of Lyapunov exponents and bifurcation diagrams with the initial state $u_{3}\left(0\right)$ are shown in Figure 8.

### 3.3. Dynamic Analysis of Circuit Parameter Variation

**Figure 9:**
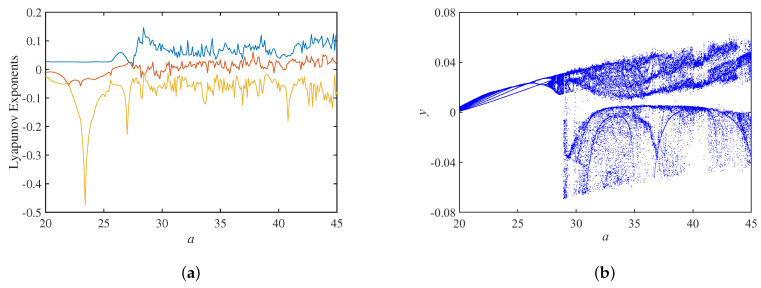
Dynamics analysis with parameter *a*: (**a**) Lyapunov exponents spectrum; (**b**) bifurcation diagram.

When setting the circuit parameters b=0.15, c=−0.2, $A_{u}=3.2$, the initial state as $\left(0.01,0,0,0\right)$, the Lyapunov exponents spectrum with the circuit parameter *a* is shown in Figure 9a, and the corresponding bifurcation diagram is shown in Figure 9b. The phase trajectory of the system is initially a periodic limit cycle, and it evolves as a single-scroll attractor at a=27 and as a double-scroll attractor at a=30. It is also observed that there are several narrow period-windows in the chaotic region.

**Figure 10:**
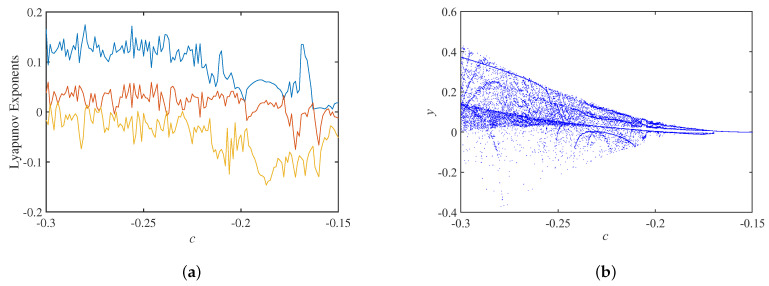
Dynamic analysis with parameter *c*: (**a**) Lyapunov exponents spectrum; (**b**) bifurcation diagram.

Setting the circuit parameters a=40, b=0.15, $A_{u}=3.2$, the initial state as $\left(0.01,\;0,\;0,\;0\right)$, the Lyapunov exponents spectrum about the circuit parameter *c* is shown in Figure 10a, and the corresponding bifurcation diagram is shown in Figure 10b. Initially, the phase trajectory of the system behaves as a double-scroll attractor, and it evolves into a limit cycle when c=−0.171 and a sink when c=−0.153.

### 3.4. Coexistence Phenomenon

**Figure 11:**
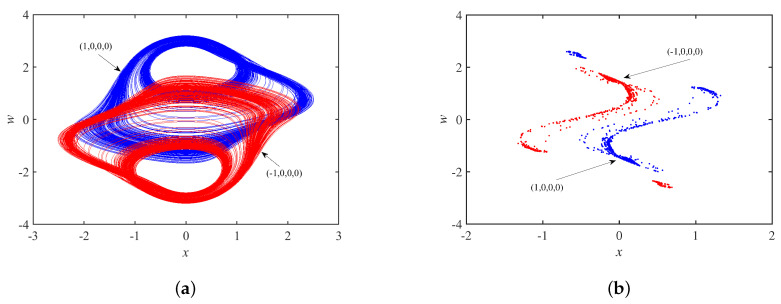
Dynamics behavior with initial state $u_{1}\left(0\right)$: (**a**) coexisting single-scroll attractors; (**b**) Poincaré maps.

**Figure 12:**
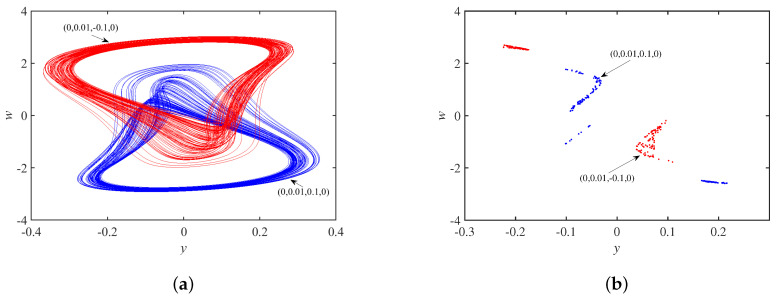
Dynamics behavior with initial state $u_{3}\left(0\right)$: (**a**) coexisting single-scroll attractors; (**b**) Poincaré maps.

Setting the parameter set *S* and the initial state as $\left(\pm 1,\;0,\;0,\;0\right)$, Figure 11a shows coexisting single-scroll attractors, and the corresponding Poincaré maps in the x−w plane is in Figure 11b, which are centrosymmetric about the origin. In other words, the system is chaotic under these conditions. Set the parameter set *S* and the initial state as $\left(0,\;0.01,\;\pm 0.1,\;0\right)$. Figure 12a shows asymmetric coexisting single-scroll attractors, and the corresponding to Poincaré maps y−w plane is in Figure 12b.

**Figure 13:**
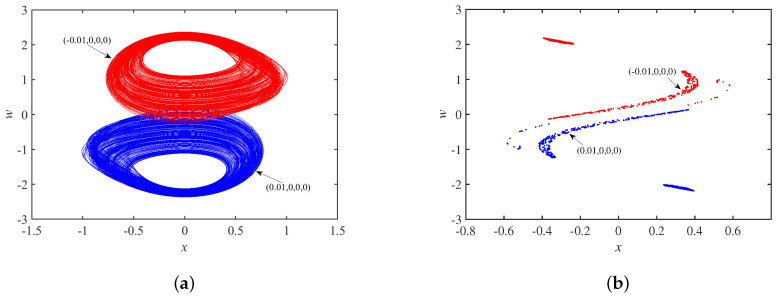
When a=29, b=0.15, c=−0.2: (**a**) coexisting single-scroll attractors; (**b**) Poincaré maps.

In Figure 13a, the phase trajectory of the system is a coexisting single-scroll attractor when the circuit parameters are set to a=29, b=0.15, c=−0.2, $A_{u}=3.2$, and the initial state is $\left(\pm 0.01,\;0,\;0,\;0\right)$. Selecting the z=0 cross section, the Poincaré maps corresponding to x−w plane are in Figure 13b, and it can be seen that the two coexisting single-scroll attractors are centrosymmetric about the origin.

**Figure 14:**
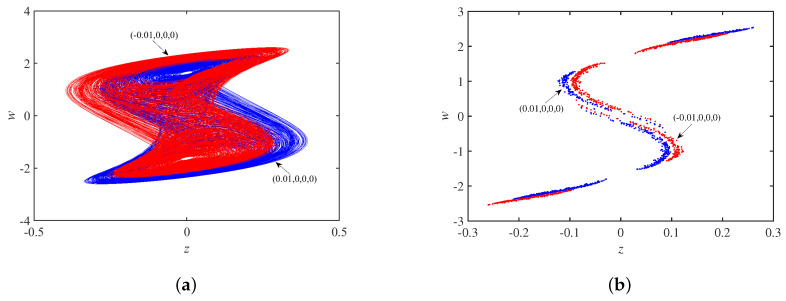
When a=33, b=0.15, c=−0.2: (**a**) coexisting double-scroll attractors; (**b**) Poincaré maps.

In Figure 14a, the phase trajectory of the system is a coexisting double-scroll attractor when the circuit parameters are set to a=33, b=0.15, c=−0.2, $A_{u}=3.2$ and the initial state is $\left(\pm 0.01,\;0,\;0,\;0\right)$. Selecting the z=0 cross section, the Poincaré maps corresponding to x−w plane is in Figure 14b, and the two coexisting double-scroll attractors are centrosymmetric about the origin.

**Figure 15:**
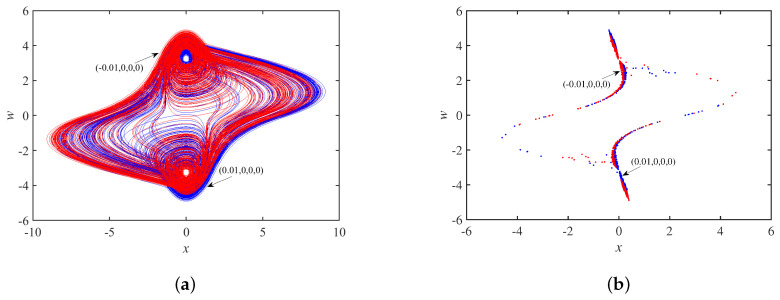
When a=40, b=0.15, c=−0.25: (**a**) coexisting double-scroll attractors; (**b**) Poincaré maps.

When the circuit parameters are set to a=40, b=0.15, c=−0.25, $A_{u}=3.2$ and the initial state is set to $\left(\pm 0.01,\;0,\;0,\;0\right)$, the phase trajectory of the system is a coexisting double-scroll attractor in Figure 15a. The y=0 cross section is selected, and the Poincaré maps corresponding to the x−w plane are in Figure 15b, which shows that the two coexisting double-scroll attractors are also centrosymmetric about the origin.

The dynamic behaviors with the change of circuit paramaters are summarized in Table 1.

## 4. The Complex Dynamical Behavior Exhibited by the Change of Initial State *φ*(0)

**Figure 16:**
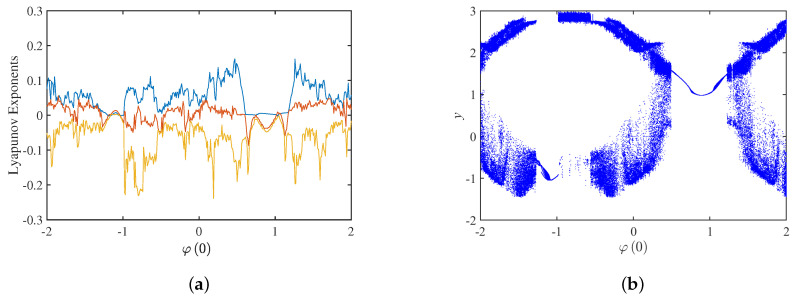
Dynamics analysis with initial state $\varphi \left(0\right)$: (**a**) Lyapunov exponents spectrum; (**b**) bifurcation diagram.

The parameters are set to a=40, b=0.15, c=−0.2, A=3.2, but initial state $\varphi \left(0\right)$ is variable. The bifurcation diagram and Lyapunov exponents spectrum with the initial state $\varphi \left(0\right)$ in the interval $\left[-2,\;\;2\right]$ are shown in Figure 16. Only three Lyapunov exponents are shown in Figure 16a, and the fourth negative Lyapunov exponent is omitted for the convenience of observation. Figure 16b shows the bifurcation diagram with $\varphi \left(0\right)$ when the z=0 cross section is selected. The bifurcation diagram is consistent with the Lyapunov exponents spectrum, and the system exhibits complex dynamical behavior as the initial state $\varphi \left(0\right)$ increases. When $\varphi \left(0\right)$ is in the interval $\left[-1.271,\;-0.982\right]$, $\left[0.493,\;1.237\right]$ and $\left[1.263,1.298\right]$, the trajectory of the system behaves as a periodic limit cycle. When $\varphi \left(0\right)$ is in the interval $\left[-2,\;-1.272\right]$, $\left[-0.981,\;-0.367\right]$, $\left[-0.366,\;0.492\right]$, $\left[1.238,\;1.240\right]$, $\left[1.299,\;1.390\right]$ and $\left[1.391,\;2\right]$, the system is chaotic. When $\varphi \left(0\right)$ is in the interval $\left[1.241,\;1.262\right]$, it is a periodic-window. The results are shown in Table 2.

**Figure 17:**
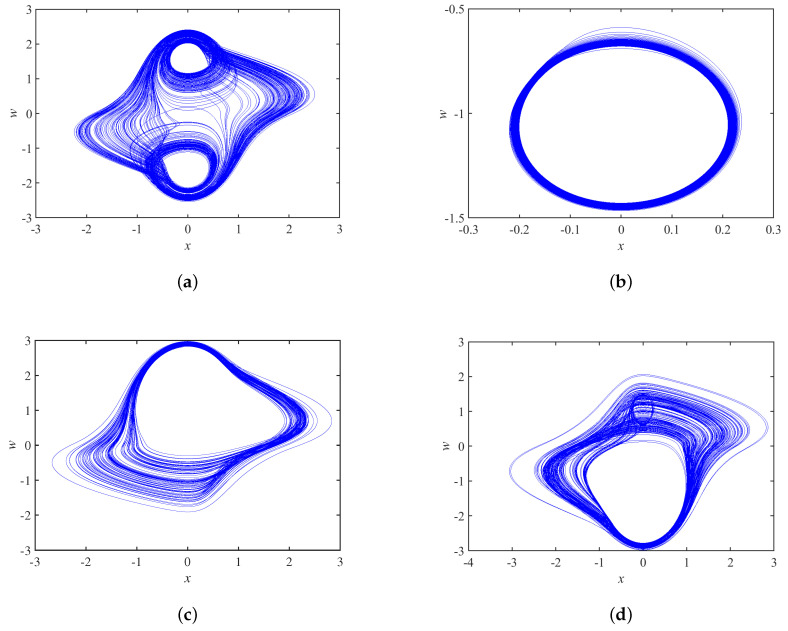
Phase portraits of initial state $\varphi \left(0\right)$ at different values on plane x−w: (**a**) $\varphi \left(0\right)$ = −1.77; (**b**) $\varphi \left(0\right)$ = −1.23; (**c**) $\varphi \left(0\right)$ = −0.98 and (**d**) $\varphi \left(0\right)$ = 1.24.

The phase trajectories with different $\varphi \left(0\right)$ are shown in Figure 17, and the system exhibits a double-scroll attractor when $\varphi \left(0\right)=-1.77$, it is a periodic limit cycle when $\varphi \left(0\right)=-1.23$, and it is a single-scroll attractor when $\varphi \left(0\right)=-0.98$, $\varphi \left(0\right)=1.24$.

**Figure 18:**
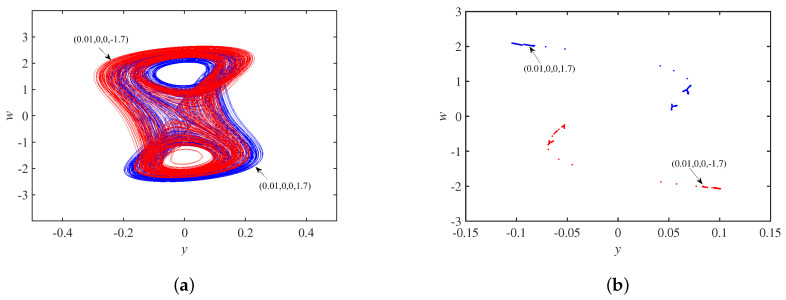
When $\varphi \left(0\right)=\pm 1.7$: (**a**) coexisting double-scroll attractors; (**b**) Poincaré maps.

**Figure 19:**
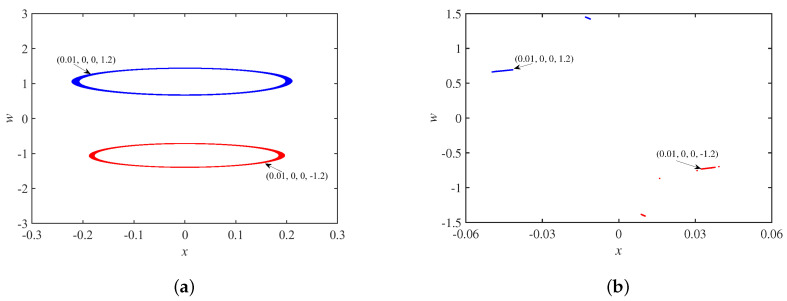
When $\varphi \left(0\right)=\pm 1.2$: (**a**) coexisting periodic limit cycle; (**b**) Poincaré maps.

Figure 18a shows the coexistence of two double-scroll attractors when the initial state $\varphi \left(0\right)=\pm 1.7$, and the corresponding Poincaré maps are in Figure 18b. Figure 19a demonstrates the coexistence of the periodic limit cycle when the initial state $\varphi \left(0\right)=\pm 1.2$, and the corresponding Poincaré maps are in Figure 19b.

## 5. Conclusions

The RC bridge oscillation chaotic circuit based on the physical memristor is proposed to fill the vacancy in the experiment teaching system of Electrical and Electronic Technology. Some dynamic behaviors of the circuit system have been researched. By changing the initial values and circuit parameters, the system exhibits the dynamical behaviors of single-scroll coexistence, double-scroll coexistence, and limit–cycle coexistence. This experiment is rigorously designed to stimulate students to expand their knowledge system and promote the application of memristor in experimental teaching.

## Figures and Tables

**Table 1:** The values of circuit parameters for various dynamical behaviors.

Values of Circuits Parameters	Dynamical Behaviors
a=29, b=0.15, c=−0.2, $A_{u}=3.2$	Coexisting single-scroll attractors
a=33, b=0.15, c=−0.2, $A_{u}=3.2$	Coexisting double-scroll attractors
a=40, b=0.15, c=−0.25, $A_{u}=3.2$	Coexisting double-scroll attractors

**Table 2:** The complex dynamical behavior with the change of Initial state $\varphi \left(0\right)$.

The Initial State	Interval	Dynamics
	$\left[-2,\;-1.272\right]$	Double-scroll attractor
	$\left[-1.271,\;-0.982\right]$	Periodic limit cycle
	$\left[-0.981,\;-0.367\right]$	Single-scroll attractor
	$\left[-0.366,\;0.492\right]$	Double-scroll attractor
$\varphi \left(0\right)$	$\left[0.493,\;1.237\right]$	Periodic limit cycle
	$\left[1.238,\;1.240\right]$	Single-scroll attractor
	$\left[1.241,\;1.262\right]$	Period-window
	$\left[1.263,\;1.298\right]$	Periodic limit cycle
	$\left[1.299,\;1.390\right]$	Single-scroll attractor
	$\left[1.391,\;2\right]$	Double-scroll attractor

## Data Availability

The data supporting the SBT memristor and the mathematical model of memristor are publicly available in two articles (Reference to [44,45]).

## Nomenclature

**Name**	**Implication**
SBT	$Sr_{0.97}Ba_{0.03}TiO_{3-\delta }$
$A_{u}$	The amplification of OP-AMP1
*a*	$\frac{C_{2}}{C_{1}}$
*b*	$\frac{R_{2}}{R_{1}}$
*c*	$\frac{R_{2}}{R_{mn}}$
$u_{1}\left(t\right)$	The voltage of the capacitor $C_{1}$
$u_{2}\left(t\right)$	The voltage of the capacitor $C_{2}$
$u_{3}\left(t\right)$	The voltage of the capacitor $C_{3}$
$\varphi \left(t\right)$	The internal flux of the SBT memristor
